# Supplementary material for: Heat adaptation of phage T7 under an extended genetic code
Source: Virus Evol. 2021 Dec 1;7(2):veab100. doi: 10.1093/ve/veab100 (PMC8923235; doi:10.1093/ve/veab100)
Supplement: veab100_Supp [file veab100_supp.zip › Sequencing_Analysis.zip!/Sequencing_Analysis/S1/evidence/RA_20.html]

BRESEQ :: Evidence


| Read alignment evidence... | | | | | | | | | | | |
| --- | --- | --- | --- | --- | --- | --- | --- | --- | --- | --- | --- |
|  | seq id | position | | ref | new | freq | score (cons/poly) | reads | annotation | genes | product |
| \* | NC\_001604.1 | 20,544 | 0 | G | A | 38.4% | 3029.7 / inf | 4188 | intergenic (–/–) | *–/–* | –/– |
| Reads supporting (aligned to +/- strand):  **ref** base G (1258/1298);  **new** base A (774/816);  **total** (2053/2135) | | | | | | | | | | | |
| Fisher's exact test for biased strand distribution *p*-value = 7.49e-01 | | | | | | | | | | | |
| Kolmogorov-Smirnov test that lower quality scores support variant *p*-value = 1.84e-01 | | | | | | | | | | | |

Only 100 of 4209 total aligned reads displayed.  

`GAGAAACGAACAGGACTTGCGGAGGATGGCGCAAAGTCTGTCTATGAGCGTTTAAAGAACGACCGTGCTCCCTATGAGACACGCGCTCAGAATTGCGCTCAATATACCATCCCATCATTGTTCCCTAAGGACTCCGATAACGCCTCTACAGATTATCAAACTCCGTGGCAAGCCGTGGGCGCTCGTGGTCTGAACAATCTAGCCTCTAAGCTCATGCTGGCTCTATTCCCTATGCAGACTTGGATGCGACTTACTATATCTGAATATGAAGCAAAGCAGTTACTGAGCGACCCCGATGGACTCGCTAAGGTCGATGAGGGCCTCTCGATGGTAGAGCGTATCATCATGAACTACATTGAGTCTAACAGTTACCGCGTGACTCTCTTTGAGGCTCTCAAACAGTTAGTCGTAGCTGGTAACGTCCTGCTGTACCTACCGGAACCGGAAGGGTCAAACTATAATCCCATGAAGCTGTACCGATTGTCTTCTTATGTGGTCCAACGAGACGCATTCGGCAACGTTCTGCAAATGGTGACTCGTGACCAGATAGCTTTTGGTGCTCTCCCTGAGGACATCCGTAAGGCTGTAGAAGGTCAAGG  >  NC_001604.1/20246‑20844`  

                                                                                                                                                                                                                                                                                                          `|`                                                                                                                                                                                                                                                                                                              
     `aCGAACAGGAATTGCGGAGGATGGCGCAAAGTCTGTCTATGAGCGTTTAAAGAACGACCGTGCTCCCTATGAGACACGCGCTCAGAATTGCACTCAATATACCATCCCATCATTGTTCCCTAAGGACTCCGATAACGCCTCTACAGATTATCAAACTCCGTGGCAAGCCGTGGGCGCTCGTGGTCTGAACAATCTAGCCTCTAAGCTCATGCTGGCTCTATTCCCTATGCAGACTTGGATGCGACTTACTATATCTGAATATGAAGCAAAGCAGTTACTGAGCGACCCCGATGAACTCGCt                                                                                                                                                                                                                                                                                                       <  1:476399/301‑1 (MQ=255)`  
         `aCAGGACTTGCGGAGGATGGCGCAAAGTCTGTCTATGAGCGTTTAAAGAACGACCGTGCTCCCTATGAGACACGCGCTCAGAATTGCGCTCAATTTACCATCCCATCATTGTTCCCTAAGGACTCCGATAACGCCTCTACAGATTATCAAACTCCGTGGCAAGCCGTGGGCGCTCGTGGTCTGAACAATCTAGCCTCTAAGCTCATGCTGGCTCTATTCCCTATGCAGACTTGGATGCGACTTACTATATCTGAATATGAAGCAAAGCAGTTACTGAGCGACCCCGATGAACTCGCTAAgg                                                                                                                                                                                                                                                                                                   <  1:341768/301‑1 (MQ=255)`  
          `aaGGACTTGCGGAGGATGGCGCAAAGTCTGTCTATGAGCGTTTAAAGAACGACCGTGCTCCCTATGAGACACGCGCTCAGAATTGCGCTCAATATACCATCCCATCATTGTTCCCTAAGGACTCCGATAACGCCTCTACAGATTATCAAACTCCGTGGCAAGCCGTGGGAGCTCGTGGTCTGAACGATCTAGCATCTAAGCTCATGCTGGCTCTATTCCCTATGCAGACTTGGATGCGACTTACTATATCTGAATATGAAGCAAAGCAGTTACTGAGCGACCCCGATGGACTCGCTAAGGt                                                                                                                                                                                                                                                                                                  <  1:11630/300‑1 (MQ=255)`  
                  `ggagatgtgtagaagagacaggtctATGAGCGTTTAAAGAACTACCGTGCTCCCTATGAGACACGCGCTCAGAATTGCGCTAAATATACCAGCCCATCATTGTTCCCGAAGGACTCCGATAACGCCTCTACAGATTATCAAACTCCGTGGCAAGCCGTGGGCGCTCGTGGTCTGAACAATCTAGCCTCTAAGCTCATGCTGGCTCTATTCCCTATGCAGACTTGGATGCGACTTACTATATCTGAATATGAAGCAAAGCAGTTACTGAGCGACCCCGATGGAATCGAGAAGGGAGaggagg                                                                                                                                                                                                                                                                                          <  1:643566/280‑1 (MQ=255)`  
                        `gaTGGCGCAAAGTCTGTCTATGAGCGTTTAAAGAACGACCGTGCTCCCTATGAGACACGCGCTCAGAATTGCGCTCAATATACCATCCCATCATTGTTCCCTAAGGACTCCGATAACGCCTCTACAGATTATCAAACTCCGTGGCAAGCCGTGGGCGCTCGTGGTCTGAACAATCTAGCCTCTAAGCTCATGCTGGCTCTATTCCCTATGCAGACTTGGATGCGACTTACTATATCTGAATATGAAGCAAAGCAGTTACTGAGCGACCCCGATGAACTCGCTAAGGTCGATGAGGGCctct                                                                                                                                                                                                                                                                                    >  1:23258/1‑301 (MQ=255)`  
                            `gagacaggtctgtctATGATCGTTTAAAGAACGACCGTGCTCCCTATGAGACACGCGCTCAGAATTGCGCTCAATATACCATCCCATCATTGTTCCCTAAGGACTCCGATAACGCCTCTACAGATTATCAAACTCCGTGGCAAGCCGTGGGCGCTCGTGGTCTGAACAATCTAGCCTCTAAGCTCATGCTGACTCTATTCCCTATGCAGACTTGGATGCGACTTACTATATCTGAATATGAAGCAAAGCAGTTACTGAGCGACCCCGATGAACTCGCTAAGGTTGATGAGGGCCTCTCGAt                                                                                                                                                                                                                                                                                <  1:318970/294‑1 (MQ=255)`  
                                 `aagcttgtcgagtaGCGTTTAAAGAAAGACCGGTCTCCCAATGAGACACGCACTAAGAATTGGGCTCAATATAACATCCCATCATTGTTCCGTAAGGACTCCGATAACGCCTCTAAAGATTATCAAACGCCGTGGCAAGCCGTGGGCGCTCGTGGTCTGAACAATCTAGCCGCTAAGCTCATGCTGGCTCTATTCCCTATGAAGACTTGGAGGCGACTTACTATATCTGAATATGAAGCAAAGCAGTTACTGAGCGACCCCGATGGACTCGCTAAGGTCGATGAGGGCCTCTCGATGGTag                                                                                                                                                                                                                                                                           <  1:649927/288‑1 (MQ=255)`  
                                   `gtctgtctATGAGCGTTTAAAGAACGACCGTGCTCCCTATGAGACACGCGCTCAGAATTGCGCTCAATATACCATCCCATCATTGTTCCCTAAGGACTCCGATAACGCCTCTACAGATTATCAAACTCCGTGGCAAGCCGTGGGCGCTAGTGGTCTGAACAATCTAGCCTCTAAGCTCATGCTGGCTCTATTCCCTATGCAGACTTGGATGCGACTTACTATATCTGAATATGAAGCAAAGCAGTTACTGAGCGACCCCGCTGGTCTCGCCAAGGTCGATGAGGGCCTCTCGATGGTagag                                                                                                                                                                                                                                                                         >  1:457454/1‑301 (MQ=255)`  
                                           `aTGAGCGTTTAAAGAACGACCGTGCTCCCTATGAGACACGCGCTCAGAATTGCGCTCAATATACCATCCCATCATTGTTCCCTAAGGACTCCGATAACGCCTCTACAGATTATCAAACTCCGTGGCAAGCCGTGGGCGCTCGTGGTCTGAACAATCTAGCCTCTAAGCTCATGCTGGCTCTATTCCCTATGCAGACTTGGATGCGACTTACTATATCTGAATATGAAGCAAAGCAGTTACTGAGCGACCCCGATGGACTCGCTAAGGTCGATGAGGGCCTCTCGATGGTAGAGCGTatcat                                                                                                                                                                                                                                                                 <  1:22604/301‑1 (MQ=255)`  
                                           `aTGAGCGTTTAAAGAACGACCGTGCTCCCTATGAGACACGCGCTCAGAATTGCGCTCAATATACCAACCCATTATTGTTCCCTAAGGACTCCGATAACTCCTCTACAGATTATCAAACTCCGTGGCAAGCCGTGGGCGCTCGTGGTCTGAACAATCTAGCCTCTAAGCTCATGCTGGCTCTATTCCCTATGCAGACTTGGATGCGACTTACTATATCTGAATATGAAGCAAAGCAGTTACTGAGCGACCCCGATGAACTCGCTAAGGTCGATGAGGGCCTCTCGATGGTAGAGCGTatcat                                                                                                                                                                                                                                                                 <  1:277786/301‑1 (MQ=255)`  
                                                `cGTTTAAAGAACGACCGTGCTCCCTATGAGACACGCGCTCAGAATTGCGCTCAATATACCATCCCATCATTGTTCCCTAAGGACTCCGATAACGCCTCTACAGATTATCAAACTCCGTGGCAAGCCGTGGGCGCTCGTGGTCTGAACAATCTAGCCTCTAAGCTCATGCTGGCTCTATTCCCTATGCCGACTTGGATGCGACTTACTATATCTGAATATGAAGCAAAGCAGTTACTGAGCGACCCCGATGAACTCGCTAAGGTCGATGAGGGCCTCTCGATGGTAGAGCGTATCATCATGa                                                                                                                                                                                                                                                            >  1:571753/1‑301 (MQ=255)`  
                                                  `tttAAAGAACGACCGTGCTCCCTATGAGACACGCGCTCAGAATTGCGCTCAATATACCATCCCATCATTGTTCCCTAAGGACTCCGATAACGCCTCTACAGATTATCAAACTCCGTGGCAAGCCGTGGGCGCTCGTGGTCTGAACAATCTAGCCTCTAAGCTCATGCTGGCTCTATTCCCTATGCAGACTTGGATGCGACTTACTATATCTGAATATGAAGCAAAGCAGTTACTGAGCGACCCCGATGGACTCGCTAAGGTCGATGAGGGCCTCTCGATGGTAGAGCGTATCATCATGAAc                                                                                                                                                                                                                                                          <  1:178065/301‑1 (MQ=255)`  
                                                  `ttgaaaGAACTAACGTGCCCCAGATCAGACACGGTCCAAGAATTGCGTTCAATATACCAACCCATCATTGTTACCTAAGGACTCCGATAACGCCTCTACAGATTAGAAAACTCCGGGGCAAGCCGTGGGCGCTCGTGGTGTGAACAATCTAGCCTCTAAGATCATGCTGGCTCTATTCCCTAGGCAGACTTGGATTCGACTTACTATATCAGAATATGAAGCAAAGCAGTTACTGAGCGACCTCGATGGACTCGCTAAGGTCGATGAGTGCCTCTCGATGGTAGAGCGTATCATCATGAAc                                                                                                                                                                                                                                                          <  1:365182/298‑1 (MQ=255)`  
                                                       `agacagACCGTGCTCCCTATGAGACACGCGCTCAGAATTGCGCTCAATATACCATCCCATCATTGTTCCCTAAGGACTCCGATAACGCCTCTACAGATTATCAAACTCCGTGGCAAGCCGTGGGCGCTCGTGGTCTGAACAATCTAGCCTCTAAGCTCATGCTGGCTCTATTCCCTATGCAGACTTGGATGCGACTTACTATATCTGAATATGAAGCAAAGCAGTTACTGAGCGACCCCGATGGACTCGCTAAGGTCGATGAGGGCCTCTCGATGGTAGAGCGTATCATCATGAACTACAt                                                                                                                                                                                                                                                     <  1:610538/296‑1 (MQ=255)`  
                                                            `gACCGTGCTCCCTATGAGACACGCGCTCAGAATTGCGCTCAATATACCATCCCATCATTGTTCCCTAAGGACTCCGATAACGCCTCTACAGATTATCAAACTCCGTGGCAAGCCGTGGGCGCTCGTGGTCTGAACAATCTAGCCTCTAAGCTCATGCTGGCTCTATTCCCTATGCAGACTTGGATGCGACTTACTATATCTGAATATGATGCAAAGCAGTTACTGAGCGACCCCGATGAACTCGCTAAGGTCGATGAGGGCCTCTCTATGGTAGAGCGTATCATCATGAACTACATTGAGt                                                                                                                                                                                                                                                <  1:654428/301‑1 (MQ=255)`  
                                                             `aCCGTGCTCCCTATGAGACACGCGCTCAGAATTGCGCTCAATATACCATCCCATCATTGTTCCCTAAGGACTCCGATAACGCCTCTACAGATTATCAAACTCCGTGGCAAGCCGTGGGCGCTCGTGGTCTGAACAATCTAGCCTCTAAGCTCATGCTGGCTCTATTCCCTATGCAGACTTGGATGCGACTTACTATATCTGAATATGAAGCAAAGCAGTTACTGAGCGACCCCGATGGACTCGCTAAGGTCGATGAGGGCCTCTCGATGGTAGAGCGTATCATCATGAACTACATTGAGtt                                                                                                                                                                                                                                               >  1:423894/1‑300 (MQ=255)`  
                                                                 `tGCTCCCTATGAGACACGCGCTCAGAATTGCGCTCAATATACCATCCCATCATTGTTCCCTAAGGACTCCGATAACGCCTATACAGATTATCAAACTCCGTGGCAAGCCGTGGGCGCTCGTGGTCTGAACAATCTAGCCTCTAAGCTCATGCTGGCTCTATTCCCTATGCAGACTTGGATGCGACTTACTATATCTGAATATGAAGCAAAGCAGTTACTGAGCGACCCCGATGGACTCGCTAAGGTCGATGAGGGCCTCTCGATGGTAGAGCGTATCATCATGAACTACATTGAGTCTAAc                                                                                                                                                                                                                                           <  1:564732/301‑1 (MQ=255)`  
                                                                   `cTCCCTATGAGACACGCACTCAGAAGTGCGCTCAATATACCATCACATCATTGTTCCCTAAGGACTCCGATAACGCCTCTACAGATTATCAAACTCCGTGGCAAGCCGTGGGCGCTCGTGGTCTGAACAATCTAGCCTCTAAGCTCATGCTGGCTCTATTCCCTATGCAGACTTGGATGCGACGTACTATATCTGAATATGAAGCAAAGCAGTTACTGAGCGACCCCGATGAACGCGCTAAGGTCGATGAGGGCCTCTCGATGGTAGAGCGTATCATCATGAACTACATTGAGTCTAACAg                                                                                                                                                                                                                                         <  1:661280/301‑1 (MQ=255)`  
                                                                       `cTATGAGACACGCGCTCAGAATTGCGCTCAATATACCATCCCATCATTGTTCCCTAAGGACTCCGATAACGCCTCTACAGATTATCAAACTCCGTGGCAAGCCGTGGGCGCTCGTGGTCTGAACAATCTAGCCTCTAAGCTCATGCTGGCTCTCTTACCTATGCCGACTTGGATGCGACTTACTATATCTGAATATGAAGCAAAGCAGTTACTGAGCGACCCCGATGAACTCGCTAAGGTCGATGAGGGCCTCTCGATGGTAGAGCGTatcactgtctcttatacacatctccgagcccac                                                                                                                                                                                                                                     >  1:436386/1‑272 (MQ=255)`  
                                                                       `cTATGAGACACGCGCTAAGAATTGCGCTCAATATACCATCCCATCATTGTTCCCTAAGGACTCCGATAACGCCTCTACAGATTATCAAACGCCGTGGCAAGCCGTGGGCGCTCGTGGTCTGAACAATCTAGCCTCTAAGCTCATGCTGGCTCTATTCCCTATGAAGACTTGGATGCGACTTACTATATCTGAATATGAAGCAAAGCAGTTACAGAGCGACCCCGATGGACTCGCTAAGGTCGATGAGGGCCTCTCGATGGTAGAGCGTATCATCATGAACTACATTGAGTCTAACAGTTAc                                                                                                                                                                                                                                     <  1:508085/301‑1 (MQ=255)`  
                                                                         `aTGAGACACGCGCTCAGAATTGCGCTCAATATACCATCCCATCATTGTTCCCTAAGGACTCCGATAACGCCTCTACAGATTATCAAACTCCGTGGCAAGCCGTGGGCGCTCGTGGTCTGAACAATCTAGCCTCTAAGCTCCTGCTGGCTCTATTCCCTATGCAGACTTGGATGCGACTTACTATATCTGAATATGAAGCAAAGCAGTTACTGAGCGACCCCGATGAACTCGCTAAGGTCGATGAGGGCCTCTCGATGGTAGAGCGTATCATCATGAACTACATTGAgctgtctcttataca                                                                                                                                                                                                                                   >  1:346388/1‑287 (MQ=255)`  
                                                                                 `cgcgCTCAGAATTGCGCTCAATATACCATCCCATCATTGTTCCCTAAGGACTCCGATAACGCCTCTACAGATTATCAAACTCCGTGGCAAGCCGTGGGCTCTCGTGGTCTGAACAATCTAGCCTCTAAGCTCATGCTGGCTCTATTCCCTATGCAGACTTGGATGCGACTTACTATATCTGAATATGAAGCAAAGCAGTTACTGAGCGACCCCGATGAACTCGCTAAGGTCGATGAGGGCCTCTCGATGGTAGAGCGTATCATCATGAACTACATTGAGTCTAACAGTTACCGCGTGActc                                                                                                                                                                                                                           <  1:262726/301‑1 (MQ=255)`  
                                                                                         `aaaTTGCGCTCAATATACCATCCCATCATTGTTCCCTAAGGACTCCGATAACGCCTCTACAGATTATCAAACTCCGTGGCAAGCCGTGGGCGCTCGTGGTCTGAACAAGCTAGCCTCTAAGATCATGCTGGCTCTATTCCCTATGCAGACTTGGATGCGACTTACTATATCTGAATATGAAGCAAAGCAGTTACTGAGCGACCCCGATGAACTCGCTAAGGTCGATGAGGGCCTCTCGATGGTAGAGCGTATCATCATGAACTACATTGAGTCTAACAGTTACCGCGTGACTCTCTTTGAg                                                                                                                                                                                                                   <  1:211540/300‑1 (MQ=255)`  
                                                                                          `aagagacagcAATATACCATCCAAACATGGTTGCCTAAGGACTCCGATAACGCATCTACAGATTATCAAACTCCGTGGCAAGGAGTGGGCGATCGTGGTCTGAACAATCTAGCCTCTAAGGTCATGCTGGATCTATTCCCTATGCAGACGTGGATGCGACTTACTATATCTGAATATGAAGCATAGCAGTTACTGAGCGACCCCGATGGACTCGCTAAGGTCGATGAGGGCCTCTCGATGGTAGAGCGTATCATCATGAACTACATTGAGTCTAACAGTTACCGCGTGACTCGCTTTGAgg                                                                                                                                                                                                                  <  1:576770/292‑1 (MQ=255)`  
                                                                                           `aTTGAGATCAATATACAATCCCATCATTGTTCCCTAAGGACTCCGATAACGCCTCTACAGATTATCAAACTCCGTGGCAAGCCGTGGGCGCTCGTGGTCTGAACAATCTAGCCTCTAAGCTCATGCTGGCTCTATTCCCTATGCAGACTTGGATGCGACTTACTATATCTGAATATGAAGCAAAGCAGTTACTGAGCGACCCCGATGGACTCGCTAAGGTCGATGAGGGCCTCTCGATGGTAGAGCGTATCATCATGAACTACATTGAGTCTAACAGTTACCGCGTGACTCTCTTTGAGGc                                                                                                                                                                                                                 <  1:490689/301‑1 (MQ=255)`  
                                                                                                   `cAATATACCATCCCATCATTGTTCCCTAAGGACTCCGATAACGCCTCTACAGATTATCAAACTCCGTGGCAAGCCGTGGGCGCTCGTGGTCTGAACAATCTAGCCTCTAAGCTCATGCTGGCTCTATTCCCTATGCAGACTTGGATGCGACTTACTATATCTGAATATGAAGCAAAGCAGTTACTGAGCGACCCCGATGGACTCGCTAAGGTCGATGAGGGCCTCTCGATGGTAGAGCGTATCATCATGAACTACATTGAGTCTAACAGTTACCGCGTGACTCTCTTTGAGGCTCTCAAAc                                                                                                                                                                                                         >  1:546717/1‑301 (MQ=255)`  
                                                                                                   `cAATATACCATCCCATCATTGTTCCCTAAGGACTCCGATAACGCCTCTACAGATTATCAAACTCCGTGGCAAGCCGTGGGCGCTCGTGGTCTGAACAATCTAGCCTCTAAGCTCATGCTGGCTCTATTCCCTATGCAGACTTGGATGCGACTTACTATATCTGAATATGAAGCAAAGCAGTTACTGAGCGACCCCGATGAACTCGCTAAGGTCGATGAGGGCCTCTCGATGGTAGAGCGTATCATCATGAACTACATTGAGTCTAACAGTTACCGCGTGACTCTCTTTGAGGCTCTCaaaa                                                                                                                                                                                                         >  1:483704/1‑300 (MQ=255)`  
                                                                                                   `cAATATACCATCCCATCATTGTTCCCTAAGGACTCCGATAACGCCTCTACAGATTATCAAACTACGTGGCAAGCCGTGGGCGCTCGTGGTCTGAACAATCTAGCCTCTAAGCTCATGCTGGCTCTATTCCCTATGCAGACTTGGATGCGACTTACTATATCTGAATATGAAGCAAAGCAGTTACTGAGCGACCCCGATGGACTCGCTAAGGTCGATGAGGGCCTCTCGATGGTAGAGCGTATCATCATGAACTACATTGAGTCTAACAGTTACCGCGTGACTCCCTTTGAGGCTCTCaaaa                                                                                                                                                                                                         >  1:268188/1‑300 (MQ=255)`  
                                                                                                             `tcccatcATTGTTCCCTAAGGACTCCGATAACGCCTCTACAGATTATCAAACTCCGTGGCAAGCCGTGGGCGCTCGTGGTCTGAACAATCTAGCCTCTAAGCTCATGCTGGCTCTATTCCCTATGCAGACTTGGATGCGACTTACTATATCTGAATATGAAGCAAAGCAGTTACTGAGCGACCCCGATGAACTCGCTAAGGTCGATGAGGGCCTCTCGATGGTAGAGCGTATCATCATGAACTACATTGAGTCTAACAGTTACCGCGTGACTCTCTTTGAGGCTCTCAAACAGTTAGTCGt                                                                                                                                                                                               <  1:541892/301‑1 (MQ=255)`  
                                                                                                               `ccatcATTGTTCCCTAAGGACTCCGATAACGCCTCTACAGATTATCAAACTCCGTGGCAAGCCGTTGGCGCTCGTGGTCTGAACAATCTAGCCTCTAAGCTCATGCTGCCTCTCTTCCCTATGCAGACCTGGATGCGACTTACTATATCTTAATATTAAGCAAAGCAGTTACTGAGCGACCCCCATGGACTCCCTAATGTCGATCAGTGCCTCTCGCTTGTAGAGCGTATCATCATGAACTACATTTAGTCTAACCGTTACCGCGTGACTCTCTTTGAGGCTCTCAAACAGTTAGTCGTAg                                                                                                                                                                                             >  1:245115/1‑301 (MQ=255)`  
                                                                                                                       `ttactcTAAGGACTCGGATAACGCCGGTACAGATAATCAAACTCCGTAGCAAGCCGTGGGAGCGCGTGGTTTGAAGAAGCTAGCCTCTAAGCTCATGCTGGCTCTATTACCTATGCAGACTTGAAGTCGACTGAATATATCTATATATGAAGCAAAGCAGTTACTGAGCGACACCGATGGACTCGCTAAGGTCGATGAGAGCCTCTCGATGGTAGAGCGTGTCATCATGAACGAAATTGAGTCGAACAGTTACCGCGTGACTCTCTTTGAGGCTCTCAAACAGTTAGTCGTAGCTGGTAAc                                                                                                                                                                                     <  1:378250/296‑1 (MQ=255)`  
                                                                                                                       `gTTCCCTAAGGACTCCGATAACGCCTCTACAGATTATCAAACTCCGTGGCAAGCCGTGGGCTCTCGTGGTCTGAACAATCTAGCCTCTAAGCTCATGCTGGCTCTATTCCCTATGCAGACTTGGATGCGACTTACTATATCTGAATATGAAGCAAAGCAGTTACTGAGCGACCCCGATGAACTCGCTAAGGTCGATGAGGGCCTCTCGATGGTAGAGCGTATCATCATGAACTACATTGAGTCTAACAGTCACCGCGTGACTCTCTTTGAGGCTCTCAAACAGTTAGTCGTAGCTGGTAAc                                                                                                                                                                                     >  1:629701/1‑301 (MQ=255)`  
                                                                                                                         `tCCCTAAGGACTCCGATAACGCCTCTACAGATTATCAAACTCCGTGGCAAGCCGTGGGCGCTCGTGGTCTGAACAATCTAGCCTCTAATCTCATGCTGGCTCTATTCCCTATGCAGACTTGGATGCGACTTACTATATCTGAATATGAAGCAAAGCAGTTACTGAGCGACCCCGATGGACTCGCTAAGGTCGATGAGGGCCTCTCGATGGTAGAGCGTATCATCATTAACTACATTGAGTCTAACAGTTACCGCGTGACTATCTTTGAGGCTCTCAAACAGTTAGTCGTAGCTGGTAACGt                                                                                                                                                                                   >  1:240179/1‑301 (MQ=255)`  
                                                                                                                         `tCCCTAAGGACTCCGATAACGCCTCTACAGATTATCAAACTCCGTGGCAAGCCGTGGGCGCTCGTGGTCTGAACAATCTAGCCTCTAAGCTCATGCTGGCTCTATTCCCTATGCAGACTTGGATGCGACTTACTATATCTGAATATGAAGCAAAGCAGTTACTGAGCGACCCCGATGGACTCGCTAAGGTCGATTAGGGCCTCTCGATTGTAGAGCGTATCATCATGAACTACATTGAGTCTAACAGTTACCGCGTGACTCCCTTTGAGGCTCTCAAACAGTTAGTCGtctgtctcttata                                                                                                                                                                                   >  1:217752/1‑289 (MQ=255)`  
                                                                                                                          `cccTAAGGACTCCGATAACGCCTCTACAGATTATCAAACTCCGTGGCAAGCCGTGGGCGCTCGTGGTCTGAACAATCTAGCCTCTAAGCTCATGCTGGCTCTATTCCCTATGCAGACTTGGATGCGACTTACTATATCTGAATATGAAGCAAAGCAGTTACTGAGCGACCCCGATGGACTCGCTAAGGTCGATGAGGGCCTCTCGATGGTAGAGCGTATCATCATGAACTACATTGAGTCTAACAGTTACCGCGTGACTCTCTTTGAGGCTATCAAACAGTTAGTcctgtctcttatacaa                                                                                                                                                                                  >  1:534416/1‑286 (MQ=255)`  
                                                                                                                               `gagaaagcGATAACGCCTCTACAGATTATCAAACTCCGTGGCAAGCCGTGGGCGCTCGTGGTCTGAACAATCTAGCCTCTAAGCTCATGCTGGCTCTATTCCCTATGCAGACTTGGATGCGACTTACTATATCTGAATATGAAGCAAAGCAGTGAATGAGCGACCCCGATGGACTCTCTAAGGTCGATGAGGGCCTCTCGATGGTAGAGCGTATCATCATGAACTACATTGAGTCTAACAGTTACCGCGTGACTCTCTTTGAGGCTCTCAAACAGTTAGTCGTAGCTGGTAACGTCctgct                                                                                                                                                                             <  1:502898/294‑1 (MQ=255)`  
                                                                                                                                `ggACTTCGATAACGCATCTACAGATTATCAAACTCCGTGGCAAGCCGTGGGCGCTCGTGGTCTGAACAATCTAGCCTCTAAGCTCATGCTGGCTCTATTCCCTATGCAGACTTGGATGCGACTTACTATATCTGAATATGAAGCAAAGCAGTTACTGAGCGACCCCGATGAACTCGCTAAGGTCGATGAGGGCCTCTCGATGGTAGAGCGTATCATCATGAACTACATTGAGTCTAACAGTTACCGCGTGACTCTCTTTGAGGCTCTCAAACAGTTAGTCGTAGCTGGTAACGTCctgctg                                                                                                                                                                            <  1:28382/301‑1 (MQ=255)`  
                                                                                                                                `ggACTCCGATAACGCCTCTACAGATTATCAAACTCCGTGGCAAGCCGTGGGCGCTCGTGGTCTGAACAATCTAGCCTCTAAGCTCATGCTGGCTCTATTCCCTATGCAGACTTGGATGCGACTTACTATATCTGAATATGAAGCAAAGCAGTTACTGAGCGACCCCGATGGACTCGCTAAGGTCGATGAGGGCCTCTCGATGGTAGAGCGTATCATCATGAACTACATTGAGTCTAACAGTTACCGCGTGACTCTCTTTGAGGCTCTCAAAAAGTTAGTCGTAGCTGGTactgtctcttat                                                                                                                                                                            >  1:452659/1‑290 (MQ=255)`  
                                                                                                                                   `cTCCGATATCGCCTCTACACATTATCAAACTCCGTGGCAAGCCGTGTGCGCTCGTGGTCTGAACAATCTAGCCTCTAAGCGCATGCTGGCTCTATTCCCTATGCAGACTTGGATGCGACTTACTATATCTGAATATGAAGCAAAGCAGTTACTGAGCGACCCCGATGGACTCGCTAAGGTCGATGAGGGCCTCTCGATGGTAGAGCGTATCATCATGAACTACATTGAGTCTAACAGTTACCGCGTGACTCTCTTTGAGGCTCTCAAACAGTTAGTCGTAGCTGGTAACGTCCTGCTGtac                                                                                                                                                                         <  1:312443/301‑1 (MQ=255)`  
                                                                                                                                   `cTCCGATAACGCCTCTACAGATTATCAAACTCCGTGGCAAGCCGTGGGCGCTCGTGGTCTGAACAATCTAGCCTATAAGCTCATGCTGGCTCTATTCCCTATGCAGACTTGGATGCGACTTACTATATCTGAATATGAAGCAAAGCAGTTACTGAGCGACCCCGATGAACTCGCTAAGGTCGATGAGGGCCTCTCGATGGTAGAGCGTATCATCATGAACTACATTGAGTCTAACAGTTACCGCGTGACTCTCTTTGAGGCTCTCAAACAGTTAGTCGTAGCTGGTAACGTCCTGCTGtac                                                                                                                                                                         <  1:154537/301‑1 (MQ=255)`  
                                                                                                                                        `aTAACGCCTCTACAGATTATCAAACTCCGTGGCAAGCCGTGGGCGCTCGTGGTCTGAACAATCTAGCCTCTAAGCTCATGCTGGCTCTATTCCCTATGCAGACTTGGATGCGACTTACTATATCTGAATATGAAGCAAAGCAGTTACTGAGCGACCCCGATGGACTCGCTAAGGTCGATGAGGGCCTCTCGATGGTAGAGCGTATCATCATGAACTACATTGAGTCTAACAGTTACCGCGTGACTCTCTTTGAGGCTCTCAAACAGTTAGTCGTAGCTGGTAACGTCCTGCTGtacctacc                                                                                                                                                                    <  1:526449/301‑1 (MQ=255)`  
                                                                                                                                            `cGCCTCTACAGATTATCAAACTCCGTGGCAAGCCGTGGGCGCTCGTGGTCTGAACAATCTAGCCTCTAAGCTCATGCTGGCTCTATTCCCTATGCAGACTTGGATGCGACTTACTATATCTGAATATGAAGCAAAGCAGTTACTGAGCGACCCCGATGAACTCGCTAAGGTCGATTAGGGCCTCTCGATGGTAGAGCGTATCATCATGCACTACATTGAGTCTAACAGTTACCGCGTGACTCTCTTTGCGGCTCTCAAACAGTTAGTCGTAGCTGGTAACTTCctgctgctgtctctttta                                                                                                                                                                >  1:67745/1‑289 (MQ=255)`  
                                                                                                                                              `ccTCTACAGATTATCAAACTCCGTGGCAAGCCGTGGGCGCTCGTGGTCTGAACAATCTAGCCTCTAAGCTCATGCTGGCTCTATTCCCTATGCAGACTTGGATGCGACTTACTATATCTGAATATGAAGCAAAGCAGTTACTGAGCGACCCCGATGAACTCGCTAAGGTCGATGAGGGCCTCTCGATGGTAGAGCGTATCATCATGAACTACATTGAGTCTAACAGTTACCGCGTGACTCTCTTTGAGGCTCTCAAACAGTTAGTCGTAGCTGGTAACGTACTGCTGTACCTaccggaacc                                                                                                                                                              >  1:272530/1‑301 (MQ=255)`  
                                                                                                                                                 `ctACAGATTATCAAACTCCGTGGCAAGCCGTGGGCGCTCGTGGTCTGAACAATCTAGCCTCTAAGCTCATGCTGGCTCTATTCCCTATGCAGACTTGGATGCGACTTACTATATCTGAATATGAAGCAAAGCAGTTACTGAGCGACCCCGATGAACTCGCTAAGGTCGATGAGGGCCTCTCGATGGTAGAGCGTATCATCATGAACTACATTGAGTCTAACAGTTACCGCGTGACTCTCTTTGAGGCTCTCAAACAGTTAGTCGTAGCTGGTAACGTCCTGCTGTACCTaccggaaccgga                                                                                                                                                           >  1:295227/1‑301 (MQ=255)`  
                                                                                                                                                  `agacagttATCAAACTCCGTGGCAAGCCGTGGGCGCTCGTGGTCTGAACAATCTAGCCTCTAAGCTCATGCTGGCTCTATTCCCTATGCAGACTTGGATGCGACTTACTATATCTGAATATGAAGCAAAGCAGTTACTGAGCGACCCCGATGAACTCGCTAAGGTCGATGAGGGCCTCTCGATGGTAGAGCGTATCATCATGAACTACATTGAGTCTAACAGTTACCGCGTGACTCTCTTTGAGGCTCTCAAACAGTTAGTCGTAGCTGGTAACGTCCTGCTGTACCTAccggaaccggaa                                                                                                                                                          <  1:590284/295‑1 (MQ=255)`  
                                                                                                                                                           `tCAAACTCCGTGGCAAGCCGTGGGCGCTCGTGGTCTGAACAATCTAGCCTCTAAGCTCATGCTGGCTCTATTCCCTATGCAGACTTGGATGCGACTTACTATATCTGAATATGAAGCAAAGCAGTTACTGAGCGACCCCGATGGACTCGCTAAGGTCGATGAGGGCCTCTCGATGGTAGAGCGTATCATCATGAACTACATTGAGTCTAACAGTTACCGCGTGACTCTCTTTGAGGCTCTCAAACAGTTAGTCGTAGCTGGTAACGTCCTGCTGTACCTACCGGAACCGGAAGGGTCAAAc                                                                                                                                                 <  1:251137/301‑1 (MQ=255)`  
                                                                                                                                                                 `cccGTGGCAAGCCGTGGGCGCTCGTGGTCTGAACAATCTAGCCTCTAAGCTCATGCTGGCTCTATTCCCTATGCAGACTTGGATGCGACTTACTATATCTGAATATGAAGCAAAGCAGTTACTGAGCGACCCCGATGGACTCGCTAAGGTCGATGAGGGCCTCTCGATGGTAGAGCGTATCATCATGAACTACATTGAGTCTAACAGTTACCGCGTGACTCTCTTTGAGGCTCTCAAACAGTTAGTCGTAGCTGGTAACGTCCTGCTGTACCTACCGGAACCGGAAGGGTCAAACTATAAt                                                                                                                                           <  1:552553/300‑1 (MQ=255)`  
                                                                                                                                                                   `cGTGGCCAGCCGTGTGCTCTCGTGGTCTGAACAATCTAGCCTCTAAGCTCATGCTGGCTCTATTCCCTATGCAGACTTGGATGCGACTTACTATATCTGCATAGTAAGCACAGCAGTTACTGAGCGACCCCAATGGACTCGCTAAGGTCGATGAGTGCCTCTCGATGGTAGAGCGTATCATCATGAACTACATTGAGTCTAACAGTTACCGCGTGACTCTCTTTGAGGCTCTCAAACAGTTAGTCGTAGCTGGTAACGTCCTGCTGTACCTACCGGAACCGGAAGGGTCAAACTATAATcc                                                                                                                                         <  1:39907/301‑1 (MQ=255)`  
                                                                                                                                                                   `cGTGGCAAGCCTTGGGCGCTCGTGGTCTGAACAATCTAGCCGCTAAGCTCATGCTGGCCCTATTCCCTATGAAGACTTGGATGCGACTTACTATATCTGAATATGAAGCAAAGCAGTTACTGAGCGACCCCGATGGACTCGCTAAGGTCGATGAGGGCCTCTCGATGGTAGAGCGTATCATCATGAACTACATTGAGTCTAACAGTTACCGCGTGACTCTCTTTGAGGCTCTCAAACAGTTAGTCGTAGCTGGTAACGTCCTGCTGTACCTACCGGAACCGGAAGGGTCAAACTATAATcc                                                                                                                                         <  1:353160/301‑1 (MQ=255)`  
                                                                                                                                                                   `cGTGGCAAGCCGTGGGCTCTCGTGGTCTGAACAATCTAGCCTCTAAGCTCATGCTGGCTCTATTCCCTATGCAGACTTGGATGCGACTTACTATATCTGAATATGAAGCAAAGCAGTTACTGAGCGACCCCGATGAACTCGCTAAGGTCGATGAGGGCCTCTCGATGGTAGAGCGTATCATCATGAACTACATTGAGTCTAACAGTTACCGCGTGACTCTCTTTGAGGCTCTCAAACAGTTAGTCGTAGCTGGTAACGTCCTGCTGTACCTACCGGAACCGGAAGGGTCAAACTATAATcc                                                                                                                                         <  1:359186/301‑1 (MQ=255)`  
                                                                                                                                                                   `cGTGGCAAGCCGTGGGCGCTCGTGGTCTGAATAATCTAGCCTCTAAGCTCATGCTGGCTCTATTCCCTATGCGGACTTGGATGCGACTTACTATATCTGAATATGAAGCAAAGCAGTTACTGAGCGACCCCGATGGACTCGCTAAGGTCGATGAGGGCCTCTCGATGGTAGAGCGTATCATCATGAACTACATTGAGTCTAACAGTTACCGCGTGACTCTCTTTGAGGCTCTCAAACAGTTAGTCGTAGCTGGTAACGTCCTGCTGTACCTACCGGAACCGGAAGGGTCAAACTATAATcc                                                                                                                                         <  1:513861/301‑1 (MQ=255)`  
                                                                                                                                                                   `cGTGGCAAGCCGTGGGCGCTCGTGGTCTGAACAATCTAGCCTCTAAGCTCATGCTGGCTCTATTCCCTATGCAGACTTGGATGCGACTTACTATATCTGAATATGAAGCAAAGCAGTTACTGAGCGACCCCGATGGACTCGCTAAGGTCGATGAGGGCCTCTCGATGGTAGAGCGTATCATCATGAACTACATTGAGTCTAACAGTTACCGCGTGACTCTCTTTGAGGCTCTCAAACAGTTAGTCGTAGCTGGTAACGTCCTGCTGTACCTACCGGAACCGGAAGGGTCAAACTATAATcc                                                                                                                                         <  1:370921/301‑1 (MQ=255)`  
                                                                                                                                                                   `cGTGGCAAGCCGTGGGCGCTCGTGGTCTGAACAATCTAGCCTCTAAGCTCATGCTGGCTCTATTCCCTATGCAGACTTGGATGCGACTTACTATATCTGAATATGAAGCAAAGCAGTTACTGAGCGACCCCGATGGACTCGCTAAGGTCGATGAGGGCCTCTCGATGGTAGAGCGTATCATCATGAACTACATTGAGTCTAACAGTTACCGCGTGACTCTCTTTGAGGCTCTCAAACAGTTAGTCGTAGCTGGTAACGTCCTGCTGTACCTACCGGAACCGGAAGGGTCAAACTATAATcc                                                                                                                                         <  1:172671/301‑1 (MQ=255)`  
                                                                                                                                                                      `ggCCAGCCGTGGGCGCTCGTGGTCTGAACAATCTAGCCTCTAAGCTCATGCTGGCTCTATTCCCTATGCAGACTTGGATGCGACTTACTATATCTGAATATGAAGCAAAGCAGTTACTGAGCGACTCCGATGGACTCGCTAAGGTCGATGAGGGCCTCTCGATGGTAGAGCGTATCATCATGAACTACATTGAGTCTAACAGTTACCGCGTGACTCTCTTTGAGGCTCTCAAACAGTTAGTCGTAGCTGGTAACGTCCTGCTTTACCTACCGGAACCGGAAGGGTCGCACTATAATccctg                                                                                                                                      >  1:496287/1‑299 (MQ=255)`  
                                                                                                                                                                      `ggCAAGCCGTGGGCGCTCGTGGTCTGAACAATCTAGCCTCTAAGCTCATGCTGGCTCTATTCCCTATGCAGACTTGGATGCGACTTACTATATCTGAATATGAAGCAAAGCAGTTACTGAGCGACCCCGATGGACTCGCTAAGGTCGATGAGGGCCTCTCGATGGTAGAGCGTATCATCATGAACTAAATTGAGTCTAACAGTTACCGCGTGACTCTCTTTGAGGCTCTCAAACAGTTAGTCGTAGCTGGTAACGTCCTGCTGTACCTCCCGGAACCGGAAGGGTCAAACTATAATcctgt                                                                                                                                      >  1:160078/1‑298 (MQ=255)`  
                                                                                                                                                                                `gggCGCTCGTGGTCTGAACAATCTAGCCTCTAAGCTCATGCTGGCTCTATTCCCTATGCAGACTTGGATGCGACTTACTATATCTGAATATGAAGCAAAGCAGTTACTGAGCGACCCCGAAGGACTCGCTAAGGTCGATGAGGGCCTCTCGATGGTAGAGCGTATCATCATGAACTACATTGAGTCTATCAGTTACCGCGTGACTCTCTTTGAGGCTCTCAAACAGTTAGTCGTAGCTGGTAACGTCCTGCTGTACCTACCGGAACCGGAAGGGTCAAACTATAATCCCATGAAGCTGTAc                                                                                                                            <  1:59632/301‑1 (MQ=255)`  
                                                                                                                                                                                        `gagacaggAACAATCTAGCCTCTAAGCTCATGCTGGCTCTATTCCCTATGCAGACTTGGATGCGACTTACTATATCTGAATATGAAGCAAAGCAGTTACTGAGCGACCCCGATGGACTCGCTAAGGTCGATGAGGGCCTCTCGATGGTAGAGCGTATCATCATGAACTACATTGAGTCTAACAGTTACCGCGTGACTCTCTTTGAGGCTCTCAAACAGTTAGTCGTAGCTGGTAACGTCCTGCTGTACCTACCGGAACCGGAAGGGTCAAACTATAATCCCATGAAGCTGTACCGATTGtc                                                                                                                    <  1:442153/294‑1 (MQ=255)`  
                                                                                                                                                                                          `ggTCTGAACAATCTAGCCTCTAAGCTCATGCTGGCTCTATTCCCTATGCAGACTTGGATGCGACTTACTATATCTGAATATGAAGCAAAGCAGTTACTGAGCGACCCCGATGAACTCGCTAAGGTCGATGAGGGCCTCTCGATGGTAGAGCGTATCATCATGAACTACATTGAGTCTAACAGTTACCGCGTGACTCTCTTTGAGGCTCTCAAACAGTTAGTCGTAGCTGGTAACGTCCTGCTGTACCTACCGGAACCGGAAGGGTCAAACTATAATCCCATGAAGCTGTACCGATTGtctt                                                                                                                  <  1:330785/301‑1 (MQ=255)`  
                                                                                                                                                                                          `ggTCTGAACAATCTAGCCTCTAAGCTCATGCTGGCTCTATTCCCTATGCACACTTGGATGCGACTTACTATATCTTAATATGAAGCAAAGCAGTTACTGAGGGACCCCGATGGACTCGCTAAGGTCGATGAGGGCCTATCTATGGTAGAGCGTATCATCATGAACTACATTGAGTCTAACAGTTACCGCGTGACTCTCTTTGAGGCTCTCAAACAGTTAGTCGTAGCTGGTAACGTCCTGCTGTACCTACCGGAACCGGAAGCGTCAAACTCTAATCCCATGAAGCTGTACCGATTGtctt                                                                                                                  >  1:104776/1‑301 (MQ=255)`  
                                                                                                                                                                                               `gAACAATCTAGCCTCTAAGCTCATGCTGGCTCTATTCCCTATGCCGACTTGGATGCGACTTACTATATCTGAATATGAAGCAAAGCAGTTACTGAGCGACCCCGATGAACTCGCTAAGGTCGATGAGTGCCTCTCGATGGTAGAGCGTATCATCATGAACTATATTGAGTCTACCAGTTACTGCGTGACTCTCTTTGAGGCTCTCACACCGTTATTAGTCGTTGTTAACGTCCTGCTGTACCTACCGGAACCGCAAGGGTAACTCTATACTCCCATGAATCTGTATCGATTGTCTTCTTAt                                                                                                             >  1:66637/1‑301 (MQ=255)`  
                                                                                                                                                                                               `gAACAATCTAGCCTCTAAGCTCATGCTGGCTCTATTCCCTATGCAGACTTGGATGCGACTTACTATATCTGAATATGAAGCAAAGCAGTTACTGAGCGACCCCGATGAACTCGCTAAGGTCGATGAGGGCCTCTCGATGGTAGAGCGTATCATCATGAACTACATTGAGTCTAACAGTTACCGCGTGACTCTCTTTGAGGCTCTCAAACAGTTAGTCGTAGCTGGTAACGTCCTGCTGTACCTACCGGAACCGGAAGGGTCAAACTATAATCCCATGAAGCTGTACCGATTGTCTTCTTAt                                                                                                             >  1:74277/1‑301 (MQ=255)`  
                                                                                                                                                                                                `agcAATCTAGCCTCTAAGCTCATGCTGGCTCTATTCCCTATGCAGACTTGGATGCGACTTACTATATCTGAATATGAAGCAAAGCAGTTACTGAGCGACCCCGATGAACTCGCTAAGGTCGATGAGGGCCTCTCGATGGTAGAGCGTATCATCATGAACTACATTGAGTCTAACAGTTACCGCGTGACTCTCTTTGAGGCTCTCAAACAGTTAGTCGTAGCTGGTAACGTCCTGCTGTACCTACCGGAACCGGAAGGGTCAAACTATAATCCCATGAAGCTGTACCGATTGTCTTCTTAtg                                                                                                            <  1:205552/299‑1 (MQ=255)`  
                                                                                                                                                                                                `aaCAATCTAGCCTCTAAGCTCATGCTGGCTCTATTCCCTATGCAGACTTGGATGCGACTTACTATATCTGAATATGAAGCAAAGCAGTTACTGAGCGACCCCGATGGACTCGCTAATGTCGATGAGGGCCTCTCGATGGTAGAGCGTATCATCATGAACTACATTGAGTCTAACAGTTACCGCGTGACTCTCTTTGAGGCTCTCAAACAGTTAGTCGTAGCTGGTAACGTCCTGCTGTACCTACCGGAACCGGAAGGGTCAAACTATAATCCCATGAAGCTGTACCGATTGTCTTCTTAtg                                                                                                            <  1:623074/301‑1 (MQ=255)`  
                                                                                                                                                                                                  `cAATCTAGCCTCTAAGCTCATGCTGGCTCTATTCCCTATGCAGACTTGGATGCGACTTACTATATCTGAATATGAAGCAAAGCAGTTACTGAGCGACCCCGATGAACTCGCTAAGGTCGATGAGGGCCTCTCGATGGTAGAGCGTATCATCATGAACTACATTGAGTCTAACAGTTACCGCGTGACTCTCTTTGAGGCTCTCAAACAGTTAGTCGTAGCTGGTAACGTCCTGCTGTACCTACCGGAACCGGAAGGGTCAAACTATAATCCCATGAAGCTGTAcctgtctcttatacacatc                                                                                                          >  1:392614/1‑284 (MQ=255)`  
                                                                                                                                                                                                   `aaTCTAGCCTCTCAGCTCATGCTGGCTCTATTCCCTATGCAGACTTGGATGCGACTTACTATATCTTAATATGAACACAAGCAGTTACTGAGCGACCCCGATGAACTCGCAAAGGTCGATGAGGTCCGATCGATGGTAGAGCGTATCATCATGAACTACATTGAGTCTAACAGTTACCGCCTGACTCTCTTTGAGGCACTCAAACAGTTAGTCGTAGCTGGTACCTGCCTGCTGTACCTACCGGACCCGGAGGTGTCAAACTATAATCCCATGACGCTGTCCCGCTTGGcttcttctttgt                                                                                                         >  1:80016/1‑295 (MQ=255)`  
                                                                                                                                                                                                        `aGCCTCTAAGCTCATGCTGGCTCTATTCCCTATGCAGACTTGGATGCGACTTACTATATCTGAATATGAAGCAAAGCAGTTACTGAGCGACCCCGATGGACTCGCTAAGGTCGATGAGGGCCTCTCGATGGTAGAGCGTATCATCATGAACTACATTGAGTCTAACAGTTACCGCGTGACTCTCTTTGAGGCTCTCAAACAGTTAGTCGTAGCTGGTAACGTCCTGCTGTACCTACCGGAACCGGAAGGGTCAAACTATAATCCCATGAAGCTGTACCGATTGTCTTCTTATGTGGTCCaa                                                                                                    <  1:121563/301‑1 (MQ=255)`  
                                                                                                                                                                                                          `ccTCTAAGCTCATGCTGGCTCTATTCCCTATGCAGACTTGGATGCGACTTACTATATCTGAATATGAAGCAAAGCAGTTACTGAGCGACCCCGATGGACTCGCTAAGGTCGATGAGGGCCTCTCGATGGTAGAGCGTATCATCATGAACTACATTGAGTCTAACAGTTACCGAGTGACTCTCTTTGAGGCTCTCAAACAGTTAGTCGTAGCTGGTAACGTCCTGCTGTACCTACCGGAACCGGAAGGGTCAAACTATAATCCCATGAAGCTGTACCGGTTGTCTTCTTATGTGGTCCaatg                                                                                                  >  1:194389/1‑299 (MQ=255)`  
                                                                                                                                                                                                                      `tGCTGGCTCTATTCCCTATGCAGACTTGGATGCGACTTACTATATCTGAATATGAAGCAAAGCAGTTACTGAGCGACCCCGATGGACTCGCTAAGGTCGATGAGGGCCTCTCGATGGTAGAACGTATCATCATGAACTACATTGAGTCTAACAGTTACCGCGTGACTCTCTTTGAGGCTCTCAAACAGTTAGTCGTAGCTGGTAACGTCCTGCTGTACCTACCGGAACCGGCAGGGTCAAACTATAATCCCATGAAGCTGTACCGATTGTCTTCTTAtgctgtctcttatacacatctccg                                                                                      >  1:582639/1‑279 (MQ=255)`  
                                                                                                                                                                                                                          `ggCTCTATTCCCTATGCAGACTTGGATGCGACTTACTATATCTGAATATGAAGCAAAGCAGTTACTGAGCGACCCCGATGAACTCGCTAAGGTCGATGAGGGCCTCTCGATGGTAGAGCGTATCATCATGAACTACATTGAGTCTAACAGTTACCGCGTGACTCTCTTTGAGGCTCTCAAACAGTTAGTCGTAGCTAGTAACGTCCTGCTGTACCTACCGGAACCGGAAGGGTCAAACTATAATCCCATGAAGCTGTACCGATTGTCTTCTTATGTGGTCCACCgctgtctcttatacaca                                                                                  >  1:13878/1‑285 (MQ=255)`  
                                                                                                                                                                                                                           `gCTCTATTCCCTATGCAGACTTGGATGCGACTTACTATATCTGAATATGAAGCAAAGCAGTTACTGAGCGACCCCGATGGACTCGCTAAGGTCGATGAGGGCCTCTCGATGGTAGAGCGTATCATCATGAACTACATTGAGTCTAACAGTTACCGCGTGACTCTCTTTGAGGCTCTCAAACAGTTAGTCGTAGCTGGTAACGTCCTGCTGTACCTACCGGAACCGGAAGGGTCAAACTATAATCCCATGAAGCTGTACCGATTGTCTTCTTATGTGGTCCAACgactgtctcttatacaca                                                                                 >  1:335945/1‑285 (MQ=255)`  
                                                                                                                                                                                                                                 `ttCACTAAGCAGACTTGGATGCGACTTACTATATCTGAATATGAAGCAAAGCAGTTACTGAGCGACCCCGATGAACTCGCTAAGGTCGAGGAGGGCCTCGCGATGGTAGAGCGTATCATCATGAATTACATTGAGTCTAACAGTTACCGCGTGACTCTCTTTGAGGCTCTCAAACAGTTAGTCGTAGCTGGTAACGTCCTGCTGTACCTACCGGAACCGGAAGGGTCAAACTATAATCCCATGAAGCTGTACCGATTGTCTTCTTATGTGGTCCAACGAGACGCATTCGGCAACGTTCTGc                                                                           <  1:386106/301‑1 (MQ=255)`  
                                                                                                                                                                                                                                   `cccTATGCAGACTTGGATGCGACTTACTATATCTGAATATGAAGCAAAGCAGTTACTGAGCGACCCCGATGGACTCGCTAAGGTCGATGAGGGCCTCTCGATGGTAGAGCGTATCATCATGAACTACATTGAGTCTAACAGTTACCGCGTGACTCTCTTTGAGGCTCTCAAACAGTTAGTCGTAGCTGGTAACGTCCTGCTGTACCTACCGGAACCGGAAGGGTCAAACTATAATCCCATGAAGCTGTACCGATTGTCTTCTTATGTGGTCCAACGAGACGCATTCGGCAACGTTCTGCaa                                                                         >  1:462261/1‑301 (MQ=255)`  
                                                                                                                                                                                                                                            `gACTTGGATTCGACTTACTATATCTGAATATGAAGCAAAGCAGTTACTGAGCGACGCCGATGGACTCGCTCAGGTCGATGAGGGCCTATCGATGGTAGAGCGTCTCATAATGAACTACATTGAGTCTAACAGGTACCGCGTGACTCTCGTTGAGGATATCCAACAGTTAGTCGTAGCTGGTATCGTCCTGCTGTACCTACCGGAACCGCAAGTGTCACACTATAATCACATGTAGCTGTACCGCTTTTCTTCTTATGTGGTCCAACGAGAAGCATTCGGCCACGTTCTGTAAATGGTGACt                                                                >  1:41051/1‑301 (MQ=255)`  
                                                                                                                                                                                                                                                   `aTGCGACTTACTAAACCTGAATATGAAGCAAAGCAGTTACTGAGCGACCCCGATGGACTCGCTAAGGTCGATGAGGGCCTCTCGATGGTAGAGCGTATCATCATGAACTACATTGAGTCTAACAGTTACCGCGTGACTCTCTTTGAGGCTCTCAAACAGTTAGTCGTAGTTGGTAACGTCCTGCTGTACCTACCGGAACGGGAAGGGTCAAACTATAATCCCATGAAGCTGTACCGATTTTCTTCTTATGTGGTCCAACGAGACGCATTCGGCAACGTTCTGCAAATGGTGACTCGTGAcc                                                         <  1:132591/301‑1 (MQ=255)`  
                                                                                                                                                                                                                                                      `cGACTTACTATATCTGAATATGAAGCAAAGCAGTTACTGAGCGACCCCGATGGACTCGCTAAGGTCGATGAGGGCCTCTCGATGGTAGAGCGTATCATCATGAACTACATTGAGTCTAACAGTTACC‑CGTGACTCTCTTTGAGGCTCTCAAACAGTTAGTCGTAGCTGGTAACGTCCTGCTGTACCTTCCGGAACCGGAAGGGTCAAACTATAATCCCATGAAGCTGTACCGATTGTCTTCTTATGTGGTCCAACGAGACGCATTCGGCAACGTTCTGCAAATGGTGACTCGTGACCAgct                                                     >  1:299464/1‑299 (MQ=255)`  
                                                                                                                                                                                                                                                        `aCTTACTATATCTGAATATGAAGCAAAGCAGTTACTGAGCGACCCCGATGGACTCGCTAAGGTCGATGAGGGCCTCTCGATGGTAGAGCGTATCATCATGAACTACATTGAGTCTAACAGTTACCGCGTGACTCTCTTTGAGGCTCTCAAACAGTTAGTCGTAGCTGGTAACGTCCTGCTGTACCTACCGGAACCGGAAGGGTCAAACTATAATCCCATGAAGCTGTACCGATTGTCTTCTTATGTGGTCCAACGAGACGCATTCGGCAACGTTCTGCAAATGGTGACTCGTGACCAGATa                                                    >  1:447088/1‑301 (MQ=255)`  
                                                                                                                                                                                                                                                          `ttACTATATCTGAATATGAAGCAAAGCAGTTACTGAGCGACCCCGATGGACTCGCTAAGGTCGATGAGGGCCTCTCGATGGTAGAGCGTATCATCATGAACTACATTGAGTCTAACAGTTACCGCGTGACTCTCTTTGAGGCTCTCAAACAGTTAGTCGTAGCTGGTAACGTCCTGCTGTACCTACCGGAACCGGAAGGGTCAAACTATAATCCCATGAAGCTGTACCGATTGTCTTCTTATGTGGTCCAACGAGACGCATTCGGCAACGTTCTGCAAATGGTGACTCGTGACCAGAGAGc                                                  <  1:257615/301‑1 (MQ=255)`  
                                                                                                                                                                                                                                                           `tACTATATCTGAATATGAAGCAAAGCAGTTACTGAGCGACCCCGATGAACTCGCTAAGGTCGATGAGGGCCTCTCGATGGTAGAGCGTATCATCATGAACTACATTGAGTCTAACAGTTACCGCGTGACTCTCTTTGAGGCTCTCAAACAGTTAGTCGTAGCTGGTAACGTCCTGCTGTACCTACCGGAACCGTAAGGGTCAAACTATAATCCCATGAAGCTGTACCGATTGTCTTCTTATGTGGTCCAACGAGACGCATTCGGCAACGTTCTGCAAATGGTGACTCGTGACCAGATAGCt                                                 >  1:100137/1‑301 (MQ=255)`  
                                                                                                                                                                                                                                                            `tcggagatgtgtataagagacaggcaGTTACTGAGCGACCCCGATGAACTCGCTAAGGTCGATGAGGGCCTCTCGATGGTAGAGCGTATCATCATGAACTACATTGAGTCTAACAGTTACCGCGTGACTCTCTTTGAGGCTCTCAAACAGTTAGTCGTAGCTGGTAACGTCCTGCTGTACCTACCGGAACCGGAAGGGTCAAACTATAATCCCATGAAGCTGTACCGATTGTCTTCTTATGTGGTCCAACGAGACGCATTCGGCAACGTTCTGCAAATGGTGACTCGTGACCAGATAGCtt                                                <  1:600728/278‑1 (MQ=255)`  
                                                                                                                                                                                                                                                             `cTATATCTGAATATGAAGCAAAGCAGTTACTGAGCGACCCCGATGGACTCGCTAAGGTCGATGAGGGCCTCTCGATGGTAGAGCGTATCATCATGAACTACATTGAGTCTAACAGTTACCGCGTGACTCTCTTTGAGGCTCTCAAACAGTTAGTCGTAGCTGGTAACGTCCTGCTGTACCTACCGGAACCGGAAGGGTCAAACTATAATCCCATGAAGCTGTACCGATTGTCTTCTTATTTGGTCCAACGAGACGCATTCGGCAACGTTCTGCAAATGGTGACTCGTGACCAGATGGCttt                                               >  1:20094/1‑301 (MQ=255)`  
                                                                                                                                                                                                                                                               `atatCTGAATATGAAGCAAAGCAGTTACTGAGCGACCCCGATGGACTCGCTAAGGTCGATGAGGGCCTCTCGATGGTAGAGCGTATCATCATGAACTACATTGAGTCTAACAGTTACCGCGTGACTCTCTTTGAGGCTCTCAAACAGTTAGTCGTAGCTGGTAACGTCCTGCTGTACCTACCGGAACCGGAAGGGTCAAACTATAATCCCATGAAGCTGTATCGATTGTCTTCTTATGTGGTCCAACGAGACGCATTCGGCAACGTTCTGCAAATGGTGACTCGTGACCAGATAGCTTTTg                                             >  1:558580/1‑301 (MQ=255)`  
                                                                                                                                                                                                                                                                   `tgtgtataagagacaggcaGTTACTGAGCGACCCCGATGAACTCGCTAAGGTCGATGAGGGCCGCTCGATGGTAGAACGTATCATCATGAACTACATTGAGTATAACAGTTACCGCGTGACTCTCTTTGAGGCTCTCAAACAGTTAGTCGTAGCTGGTAACGTCCTGCTGTGCCTACCGGAACCGGAAGGGTCAAACTATAATCCCATGAAGCTGTACCGATTGTCTTCTTATGTGGTCCAACGAGACGCATTCGGCAACGTACTGCAAATGGTGACTCGTGACAAGATAGCTTTTGGTGc                                         <  1:429743/285‑1 (MQ=255)`  
                                                                                                                                                                                                                                                                   `cttaaTATGAAGCAAAGCAGTTACTGAGCGACCCCGAGCAACTCGCTAAGGTCGATGATGGCCTCTCGATGGTAGAGCGTATCATCATGAACTACATTGAGTCTAACAGTTACCGCGTGACTCTCTTTTAGGCTCTCAAACAGTTAGTCGTAGCTGGTAACGTCCTGCTTTACCTACCGGAACCGGAAGGGTCAAACTATAATCCCATGATGCTGTACCGATTGTCTTCTTATGTGGTCCAACGAGACGCATTCGGCAACGTTCTGCAAATGGTGACTCGTGACCAGATAGCTTTTGGTGc                                         <  1:126766/298‑1 (MQ=255)`  
                                                                                                                                                                                                                                                                       `atatGAAGCAAAGCAGTTACTGAGCGACCCCGATGGACTCGCTAAGGTCGATGAGGGCCTCTCGATGGTAGAGCGTATCATCATGAACTACTTTGAGTCTAACAGTTACCGCGTGACTCTCTTTGAGGCTCTCAAACAGTTAGTCGTAGCTGGTAACGTCCTGCTGTACCTACCGGAACCGGAAGGGTCAAACTATAATCCCATGAAGCTGTACCGATTGTCTTCTTATGTGGTCCAACGAGACTCATTCGGCAACGTTCTGCAAATGGTGACTCGTGACCAGATAGCTTTTGGTGCtctc                                     >  1:616828/1‑301 (MQ=255)`  
                                                                                                                                                                                                                                                                            `aagtaaagcaGTTACTGAGCGACCCCGATGGACTCGCTAAGGTCGATGAGGGCCTCTCGATGGTAGAGCGTATCATCATGAACTACATTGAGTCTAACAGTTACCGCGTGACTCTCTTTGAGGTTCTCAAACAGTTAGTCGTAGCTGGTAACGTCCTGCTGTACCTACCGGAACCGGAAGGGTTAACCTATACTCACATGAAGCTGTACCGCTTGTATTCTTATTTGGTCCAACGTACAGCATTCGGCAACGTTCTGCAAATGGTGACTCGTGACCAGATAGCtttatgtctcttatactc                                >  1:92156/1‑286 (MQ=255)`  
                                                                                                                                                                                                                                                                            `aagcaaagcaGTTACTGAGCGACCCCGATGAACTCGCTAAGGTCGATGAGGGCCTCTCGATGGTAGAGCGTATCATCATGAACTACATTGAGTCTAACAGTTACCGCGTGACTCTCTTTGAGGCTCTCAAACAGTTAGTCGTAGCTGGTAACGTCCTGCTGTACCTACCGGAACCGGAAGGGTCAAACTATAATCCCATGAAGCTGTACCGATTGTCTTCTTATGTGGTCCAACGAGACGCATTCGGCAACGTTCTGCAAATGGTGACTCGTGACCGGATAGCTTTTGGTGCTCTCCCTGa                                >  1:189652/1‑301 (MQ=255)`  
                                                                                                                                                                                                                                                                              `gcaaagcaGTTACTGAGCGACCCCGATGGACTCGCTAAGGTCGATGAGGGCCTCTCGATGGTAGAGCGTATCATCATGAACTACATTGAGTCTAACAGTTACCGCGTGACTCTCTTTGAGGCTCTCAAACAGTTAGTCGTAGCTGGTAACGTCCTGCTGTACCTACCGGAACCGGAAGGGTCAAACTATAATCCCATGAAGCTGTACCGATTGTCTTCTTATGTGGTCCAACGAGACGCATTCGGCAACGTTCTGCAAATGGTGACTCGTGACCAGATAGCTTTTGGTTCTCTCCCTGAgg                              >  1:86656/1‑301 (MQ=255)`  
                                                                                                                                                                                                                                                                              `gcaaagcaGTTACTGAGCGACCCCGATGGACTCGCTAAGGTCGATGAGGGCCTCTCGATGGTAGAGCGTATCATCATGAACTACATTGAGTCTAACAGTTACCGCGTGACTCTCTTTGAGGCTCTCAAACAGTTAGTCGTAGCTGGTAACGTCCTGCTGTACCTACCGGAACCGGAAGGGTCAAACTATAATCCCATGAAGCTGTACCGATTGTCTTCTTATGTGGTCCAACGAGACGCATTCGGCAACGTTCTGCAAATGGTGACTCGTGACCAGATAGCTTTTGGTGCTCTCCCTGAgg                              <  1:418054/301‑1 (MQ=255)`  
                                                                                                                                                                                                                                                                               `caacgcaGTTCCTGAGCGACCCCGAGGAACTCGCTAAGGTCCATGAGGGCCTCTCGATGTTAGTGCGTATCATCATGAACCCCATTGGGTCTAACAGTTACCGCGTGACTCTCTTTGAGGCTCTCAAATAGTTAGTCGTAGCTGGTAACGTCCTGCTGTACCTACCGCAACCGGAAGTGTCAAACTATAATCCCATGAAGCTGTACCGATTGTCTTCTTATGTGGTCCAACGAGACGCATTCGGCAACGTTCTGCAAATGGTGACTCGTGAACAGATAGCTTTTGGTGCTCTCCCTGAGGa                             <  1:520056/301‑1 (MQ=255)`  
                                                                                                                                                                                                                                                                               `caaagcaGTTACTGAGCGACCCCGATGGACTCGCTAAGGTCGATGAGGGCCTCTCGATGGTAGAGCGTATCATCATGAACTACATTGAGTCTAACAGTTACCGCGTGACTCTCTTTGAGGCTCTCAAACAGTTAGTCGTAGCTGGTAACGTCCTGCTGTACCTACCGGAACCGGAAGGGTCAAACTATAATCCCATGAAGCTGTACCGATTGTCTTCTTATGTGGTCCAACGAGACGAATTCGGCAACGTTCTGCAAATGGTGACTCGTGACCAGATAGCTTTTGGTGCTCTCCCTGAGGa                             <  1:290364/301‑1 (MQ=255)`  
                                                                                                                                                                                                                                                                               `caaagcaGTTACTGAGCGACCCCGATGGACTCGCTAAGGTCGATGAGGGCCTCTCGATGGTAGAGCGTATCATCATGAACTACATTGAGTCTAACAGTTACCGCGTGACTCTCTTTGAGGCTCTCAAACAGTTAGTCGTAGCTGGTAACGTCCTGCTGTACCTACCGGAACCGGAAGGGTCAAACGATAATCCCATGAAGCTGTACCGATTGTCTTCTTATGTGGTCCAACGAGACGCATTCGGCAACGTTCTGCAAATGGTGACTCGTGACCAGATAGCTTTTGGTGCTCTCCCTGAGGa                             <  1:146273/301‑1 (MQ=255)`  
                                                                                                                                                                                                                                                                               `aaaagcaGTTACTGGGCGACCCCGCTGAACGCGCTAAGGTCGATGAGGGCCTCTCGATGGTAGAGCGTATCATCATGAACTACATTGAGTCTAACAGTTACCGCGTGACTCTCTTTGAGGCTCTCAAACAGTTAGTCGTAGCTGGTAACGTCCTGCTGTACCTACCGGAACCGGAAGGGTCAAACTATAATCCCATGAAGCTGTACCGATTGTCTTCTTATGTGGTCCAACGAGACGCATTCGGCAACGTTCTGCAAATGGTGACTCGTGACCAGATAGCTTTTGGTGCTCTCCCTGAGGa                             <  1:468378/300‑1 (MQ=255)`  
                                                                                                                                                                                                                                                                               `aaaagcaGTTACTGAGCGACCCCGAGGGACTCGCTAAGGTCGATGAGGGCCTCTCGATGGTAGAGCGTATCATCATGAACTACATTGAGTCTAACAGTTACCGCGTGACTCTCTTTGAGGCTCTCAAACAGTTAGTCGTAGCTGGTAACGTCCTGCTGTACCTACCGGAACCGGAAGGGTCAAACTATAATCCCATGAAGCTGTACCGATTGTCTTCTTATGTGGTCCAACGAGACGCATTCGGCAACGTTCTGCAAATGGTGACTCGTGACCAGATAGCTTTTGGTGCTCTCCCTGAGGa                             <  1:404032/300‑1 (MQ=255)`  
                                                                                                                                                                                                                                                                                 `aagcaGTTACTGAGCGACCCCGATGGACTCGCTAAGGTCGATGAGGGCCTCTCGATGGTAGAGCGTATCATCATGAACTACATTGAGTCTAACAGTTACCGCGTGACTCTCTTTGAGGCTCTCAAACAGTTAGTCGTAGCTGGTAACGTCCTGCTGTACCTACCGGAACCGGAAGGGTCAAACTATAATCCCATGAAGCTGTACCGATTGTCTTCTTATTTGGTCCACCGAGACGCATTCGGCAACGTTCTGCAAATGGTGACTCGTGACCAAATATCTTTTGGTGCTATCCCTGAGGAcc                           >  1:305654/1‑300 (MQ=255)`  
                                                                                                                                                                                                                                                                                   `gcaGTTACTGAGCGACCCCGATGGACGCGCTAAGGTCGATGAGGGACTCTCGATGGTAGAGCGTATCATCATGAACTACATTGAGTCTAACAGTTACCGCGTGACTCTCTTTGAGGCTCTCAAACAGTTAGTCGTAGCTGGTAACGTCCTGCTGTACCTACCGGAACCGGAAGGGTCAAACTATAATCCCATGAAGCTGTACCGATTGTCTTCTTATGTGGTCCAACGAGACGCATTCGGCAACGTTCTGCAAATGGTGACTCGTGACCAGATAGCTTTTGGTGCTCTCCCTGAGGACATc                         <  1:110367/301‑1 (MQ=255)`  
                                                                                                                                                                                                                                                                                      `gTTACTGAGCGACCCCGATGAACTCGCTAAGGTCGATGAGGGCCTCTCGATGGTAGAGCGTATCATCATGAACTACATTGAGTCTAACAGTTACCGCGTGACTCTCTTTGAGGCTCTCAAACAGTTAGTCGTAGCTGGTAACGTCCTGCTGTACCTACCGGAACCGGAAGGGTCAAACTATAATCCCATGAAGCTGTACCGATTGTCTTCTTATGTGGTCCAACGAGACGCATTCGGCAACGTTCTGCAAATGGTGACTCGTGACCAGATAGCTTTTGGTGCTCTCCCTGAGGACATCCgg                      >  1:1842/1‑300 (MQ=255)`  
                                                                                                                                                                                                                                                                                           `tGAGCGACCCCGATGAACTCGCTAAGGTCGATGAGGGCCTCTCTATAGTAGAGCGTATCATCATGAACTACATTGAGTCTAACAGTTACCGCGTGACTCTCTTTGAGGCTCTCAAACAGTTAGTCGTAGCTGGTACCGTCCTGCTGTACCTACCGGAACCGGAAGGGTCAAACTATAATCCCATGAAGCTGTACCGATTGTCTTCTTATGTGGTCCAACGAGCCGCATTCGGCAACGTTCTGCAAATGGTGACTCGTGACCAGATAGCTTTTGGTGCTCTCCCTGAGGACATCCGTAAGGc                 <  1:324695/301‑1 (MQ=255)`  
                                                                                                                                                                                                                                                                                           `tGAGCGACCACGCTGGACTCGCTAAGGTAGATGAGGGCCTCTCGATGGTAGAGCGTATCATCATGAACTACATTGAGTCTAACAGTTACCGCGTGACTCTCTTTGAGGCTCTCAAACAGTTAGTCGTAGCTGGTAACGTCCTGCTGTACCTACCGGAACCGGAAGGGTCAAACTATAATCCCATGAAGCTGTACCGATTGTCTTCTTATGTGGTCCAACGAGACGCATTCGGCAACGTTCTGCAAATGGTGACTCGTGACCAGATAGCTTTTGGTGCTCTCCCTGAGGACATCCGTAAGGc                 <  1:636419/301‑1 (MQ=255)`  
                                                                                                                                                                                                                                                                                            `gAGCGACCCCGATGAACTCGCTAAGGTCGATGAGGGCCTCTCGATGGTAGAGCGTATCATCATGAACTACATTGAGTCTAACAGTTACCGCGTGACTCTCTTTGAGGCTCTCAAACATTTAGTCGTAGCTGGTAACGTCCTGCTGTACCTCCCGGAACCGGAAGGGTCAAACTATAATCCCATGAAGCTGTACCGATTGTCTTCTTATTTGTTCCAACGCGCCGCATTCGGCAACGTTCTGCAAATGGTGACTCGTGACCAGATAGCTTTTGGTGCTATCCCTGAGGACATCCGTAAGGCt                >  1:165415/1‑301 (MQ=255)`  
                                                                                                                                                                                                                                                                                                     `cGATGAACTCGCTAAGGTCGATGAGGGCCTCTCGATGGTAGAGCGTATCATCATGAACTACATTGAGTCTAACAGTTACC‑CGTGACTCTCTTTGAGGCTCTCAAACAGTTAGTCGTAGCTGGTAACGTCCTGCTGTACCTACCGGAACCGGAAGGGTCAAACTATAATCCCATGAAGCTGTACCGATTGTCTTCTTATGTGGTCCAACGAGACGCATTCGGCAACGTTCTGCAAATGGTGACTCGTGACCAGATAGCTTTTGGTGCTCTCCCTGAGGACATCCGTAAGGCTGTAGAAGGTc      >  1:605953/1‑301 (MQ=255)`  

                                                                                                                                                                                                                                                                                                          `|`                                                                                                                                                                                                                                                                                                              
`GAGAAACGAACAGGACTTGCGGAGGATGGCGCAAAGTCTGTCTATGAGCGTTTAAAGAACGACCGTGCTCCCTATGAGACACGCGCTCAGAATTGCGCTCAATATACCATCCCATCATTGTTCCCTAAGGACTCCGATAACGCCTCTACAGATTATCAAACTCCGTGGCAAGCCGTGGGCGCTCGTGGTCTGAACAATCTAGCCTCTAAGCTCATGCTGGCTCTATTCCCTATGCAGACTTGGATGCGACTTACTATATCTGAATATGAAGCAAAGCAGTTACTGAGCGACCCCGATGGACTCGCTAAGGTCGATGAGGGCCTCTCGATGGTAGAGCGTATCATCATGAACTACATTGAGTCTAACAGTTACCGCGTGACTCTCTTTGAGGCTCTCAAACAGTTAGTCGTAGCTGGTAACGTCCTGCTGTACCTACCGGAACCGGAAGGGTCAAACTATAATCCCATGAAGCTGTACCGATTGTCTTCTTATGTGGTCCAACGAGACGCATTCGGCAACGTTCTGCAAATGGTGACTCGTGACCAGATAGCTTTTGGTGCTCTCCCTGAGGACATCCGTAAGGCTGTAGAAGGTCAAGG  >  NC_001604.1/20246‑20844`

  

Alignment Legend

Aligned base mismatch/match (shaded by quality score): `ATCG/ATCG` `< 3 ≤` `ATCG/ATCG` `< 10 ≤` `ATCG/ATCG` `< 21 ≤` `ATCG/ATCG` `< 34 ≤` `ATCG/ATCG` `< 38 ≤` `ATCG/ATCG`

Unaligned base: `atcg`    Masked matching base: `atcg`    Alignment gap: `‑`    Deleted base: `‑`
